# State-of-the-art for automated machine learning predicts outcomes in poor-grade aneurysmal subarachnoid hemorrhage using routinely measured laboratory & radiological parameters: coagulation parameters and liver function as key prognosticators

**DOI:** 10.1007/s10143-025-03450-z

**Published:** 2025-03-17

**Authors:** Ali Haider Bangash, Jayro Toledo, Muhammed Amir Essibayi, Neil Haranhalli, Rafael De la Garza Ramos, David J. Altschul, Stavropoula Tjoumakaris, Reza Yassari, Robert M. Starke, Redi Rahmani

**Affiliations:** 1 Hhaider5 Research Group, Rawalpindi, PB, Pakistan; 2 Spine Tumor Mechanics and Outcomes Research (TUMOR) Lab, Montefiore Medical Center, Albert Einstein College of Medicine, Bronx, NY USA; 3 Department of Neurosurgery, University of Miami, Miami, FL USA; 4 Montefiore-Einstein Cerebrovascular Research Lab, Montefiore Medical Center, Albert Einstein College of Medicine, Bronx, NY USA; 5 Department of Neurosurgery, Montefiore Medical Center, Albert Einstein College of Medicine, Bronx, NY USA; 6 Department of Neurosurgery, Thomas Jefferson University Hospital, Philadelphia, PA USA; 7 Department of Neurosurgery, University of Pennsylvania, Philadelphia, PA USA

**Keywords:** Aneurysmal subarachnoid hemorrhage, Machine learning, Modified Rankin scale, Liver function, Coagulation, Logistic regression, Decision tree

## Abstract

The objective of this study was to develop and evaluate automated machine learning (aML) models for predicting short-term (1-month) and medium-term (3-month) functional outcomes [Modified Rankin Scale (mRS)] in patients suffering from poor-grade aneurysmal subarachnoid hemorrhage (aSAH), using readily available and routinely measured laboratory and radiological parameters at admission. Data from a pilot non-randomized trial of 60 poor-grade aSAH patients (Hunt-Hess grades IV or V) were analyzed. Patients were evenly divided between targeted temperature management (TTM) and standard treatment groups. The current state-of-the-art for aML was adopted to employ nine ML algorithms with hyperparameter tuning to develop algorithmic models predicting 1 month and 3-months mRS scores. Model performance was evaluated using macro-weighted average Area Under the Receiver Operating Curve (mWA-AUROC) analysis and additional metrics. Logistic regression algorithmic models achieved perfect prediction (mWA-AUROC = 1, accuracy = 100%, sensitivity and specificity = 100% [95% CI: 83.16 − 100%]) for both 1-month and 3-month mRS outcomes. For 1-month outcomes, neutrophil count, platelet count, and gamma-glutamyl transferase levels were identified as key predictors. For 3-month outcomes, patient gender, activated partial thromboplastin time, and serum aspartate aminotransferase levels were most impactful. Decision tree algorithms (mWA-AUROC = 0.9-0.925) identified specific cut-points for various parameters, providing actionable information for clinical decision-making. Positive prognostic factors included alkaline phosphatase levels higher than mid-value of their normal range, absence of hydrocephalus, use of targeted temperature management (TTM), and specific cut-offs for coagulation and liver function parameters. The use of TTM was reinforced as a key prognosticator of mRS outcomes at both time points. We have made our developed models and the associated architecture available at GitHub. This study demonstrated the potential of aML in predicting functional outcomes for poor-grade aSAH patients. The identification of novel predictors, including liver function and coagulation parameters, opens new avenues for research and intervention. While the perfect predictive performance warrants cautious interpretation and further validation, these models represent a step towards personalized medicine in aSAH management, potentially improving prognostication and treatment strategies.

## Introduction

Aneurysmal subarachnoid hemorrhage (aSAH) poses a major challenge in the domain of neurocritical care with an incidence ranging from 6.1 to 10.2 per 100,000 person-years and a global crude incidence of 6.67 per 100,000 persons, coupled with a plethora of complications including but not limited to mortality and vasospam [1–4]. Hospital death rates arereported to vary from 1.7 to 4.1%, with a significant number of deaths from complications including intracranial hypertension and delayed cerebral ischemia (DCI) [5]. Patients with poor-grade aSAH, defined as Hunt-Hess (HH) grades IV or V, continue to suffer from higher all-cause mortality, with rates varying from 24 to 36% and vasospasm-associated morbidity rates as high as 28%, despite improvements in neurocritical care protocols [4, 6, 7]. There is currently a discourse amongst healthcare professionals managing aSAH about the best course of care for these critically ill individuals, and multiple therapeutic approaches are being investigated to improve their prognosis [8].

Targeted temperature management (TTM) is a promising neuroprotective strategy adopted in a variety of acute neurological conditions, such as cardiac arrest and traumatic brain injury [9, 10]. TTM is a treatment strategy where, after an initial injury, careful regulation of a patient’s body temperature is ensured, usually by causing moderate hypothermia. This prevents further damage to the brain [11]. Its effectiveness in managing individuals with poor-grade aSAH, however, is still debated and requires further research [12]. The intricate pathophysiology of aSAH, which includes systemic challenges, DCI, and early brain damage, creates unique challenges in terms of prognostication and provision of optimized care for individual patients [13, 14].

Accurate prognostication of poor-grade aSAH is crucial for well-informed clinical judgment, prudent resource distribution, and effective counseling for families [15]. Particularly in the most serious cases, conventional scoring methods and clinical markers have shown limited validity in forecasting the course of events [16]. By leveraging intricate interactions between several factors, advanced machine learning (ML) approaches provide novel opportunities for developing models for prognostication that are more accurate and nuanced [17]. Automated ML (aML) is considered a ‘super-specialty’ within ML space where all the steps of model development from data cleaning, and feature selection to hyperparameter tuning are undertaken in an automated manner and has consistently outperformed conventional ML and conventional statistical analytical techniques in healthcare research [18, 19].

Therefore, in order to develop a robust predictive model that appreciates the nuanced interactions between parameters, we explored the statistical capabilities of aML to predict the functional outcomes of poor-grade aSAH patients over the short-term (1 month) and medium-term (3 months) time frames. Our approach blended the analytical strength of cutting-edge ML techniques with the methodological rigor of a controlled trial. With an emphasis on easily accessible and routinely measured laboratory and radiological parameters obtained at admission, our goal was to develop pragmatic prognostic models. We also looked at how TTM affected these results, which could have provided important information about its effectiveness and helped us pinpoint patient subgroups that would benefit the most from this intervention.

## Materials and methods

Data from a pilot non-randomized controlled trial exploring TTM in poor-grade aSAH patients (HH grades IV or V) were analyzed. Sixty patients were evenly divided between TTM and standard treatment groups [20]. The study was carried out following the guidelines delineated under the principles of Helsinki including respecting the articles pertinent to patient consent. Since this is a post-hoc analysis of a published study that shared de-identified data [21], approval from an institutional review board was not sought. The TRIPOD Checklist for Prediction Model Development and Validation was adopted to report the findings of our study [22]. 

### Dataset

The dataset included a wide range of easily measured clinical indicators that are often applied in the practice of neurocritical care [21]. These variables were either dichotomous or continuous. Dichotomous variables included demographic characteristics (Gender, smoking history, drinking history, stroke history, hypertension history, diabetes history and coronary disease history), radiological parameters (Aneurysm location, cerebral edema, midline shift and hydrocephalus) as well as Modified Rankin Scale (mRS) outcomes at 1 month and 3 months (Good outcomes = mRS 0–3; Bad outcomes = mRS 4–6) [21]. Continuous variables included age and routinely measured laboratory parameters on admission such as complete blood count parameters such as red platelet counts and neutrophil-to-lymphocyte ratio, liver function parameters such as GPT and alkaline phosphatase (ALP) levels, coagulation parameters such as PT and aPTT, as well as renal function parameter parameter (Creatinine level) [21]. 

### Statistical analysis

By using Python programming language in the Google Colaboratory environment, the current SOTA for aML [23] — MLjar (version 1.1.9)— was adopted to develop algorithmic models that could predict 1-month and 3-months mRS outcomes in the said patient population. The MLjar library incorporated 9 algorithms detailed hereinafter. The dataset was loaded as a.CSV file with the carageorical variables as well as the outcome handled as binary data points (ZERO and ONE), whereas the continuous variables were handled as integers. The ‘compete’ mode was chosen with the ‘explain_level’ kept at 2 (the maximum value) that allowed for the development of learning curves, importance plots and SHaP value plots, along with optimized tuning of hyper-parameters.

The first step in the development pipeline was to obtain preliminary insights into the dataset by implementing the simplest of algorithms. For ‘compete’ mode, Decision Trees algorithm was implemented which provided a simple decision rules tree with a maximum depth of 4 levels. The tree could be visualized via the ‘dtreeviz’ package. The second step was to concurrently develop models by individually adopting 7 algorithms including Extra Trees, Nearest Neighbors, eXtreme Gradiant Boosting (xGboost), Light Gradiant Boosting Machine (LGBM), Neural Network (NN), Categorical Boosting (CatBoost) and Random Forest (RF)— each algorithmic model trained with default hyperparameters. For this step, one model each was developed by adopting each one of the 7 algorithms with each algorithm having a single set of default hyperparameter values for the prediction task (binary classification), independent of the loaded dataset.

The third step was to undertake a random search over defined set of individual hyperparameters for each one of the above mentioned 7 algorithms. The hyperparameters optimized for each one of the algorithms can be explored at https://github.com/mljar/mljar-supervised/tree/master/supervised/algorithms. For the fourth step, the individual hyperparameters of best performing models for xGboost, LGBM and CatBoost algorithms obtained on second and third steps were adopted to develop an algorithmic model each for these 3 algorithms where ‘golden features’ were developed by employing arithmetic functions on variables from the original dataset in a bid to enhance the predictive capability of the resulting algorithmic models.

The fifth step marked the initiation of feature selection is further broken down into two sub-steps. During the ‘random_feature’ sub-step, a new variable with uniform distribution of positive and negative outcomes was introduced into the dataset. The extended dataset was adopted to train the algorithmic model outperforming all other models so far with its specific hyperparameters noted. A permutation-based feature importance graph was plotted to compare the performance of each variable of the original dataset against the randomly inserted novel variable in the extended dataset. The original variable was dropped if it not perform well comparatively in at least half of the total 10 learners. During the ‘features_selection’ sub-step, the respective best performing algorithmic model each for 6 algorithms (Extra Trees, Nearest Neighbors, xGboost, LGBM, NN, CatBoost and RF) with the respective hyperparameters was trained using only selected features.

Fine tuning of the algorithmic models was undertaken as the sixth step where two ‘hill_climbing’ sub-steps were undertaken to further refine models. During each ‘hill_climbing’ step, one randomly selected hyperparameter for each algorithmic model was fine tuned with a variation introduced in its respective settings in both directions. It is impressed upon that the steps 2–6, which have been dissected empirically, were infact undertaken in parallel and not longitudinally.

For the seventh step, all the developed algorithmic models were ensembled by calculating the respective weight values. For the eighth step, the prediction values obtained upon training algorithmic models on the original dataset were added to the said dataset, leading to the generation of further extended dataset. The predictions of algorithmic models obtained upon training on the novel, extended dataset, along with the original dataset, were then fed into a meta-learner— stacked model— in a bid to improve accuracy. For the last step, an ensemble stacking was undertaken to combine the ensemble developed from algorithmic models trained on the original dataset, and stacked model which was trained on the extended dataset including original dataset and stacked predictions [24]. 

Five-fold stratified, shuffled cross-validation was implemented for internal validation. Macro-weighted average Area Under the Receiver Operating Curve (mWA-AUROC) analysis was carried out to interpret the discriminating classification ability of the developed models in accordance with the schema outlined by Lau L et al. [25] Additional performance metrics such as accuracy, sensitivity, specificity, logloss, precision, recall, F1-score, and Matthews Correlation Coefficient (MCC) were also considered. The developed algorithmic models were made available online as a Google Colab notebook at GitHub.

## Results

The dataset comprised of 60 patients with poor-grade aSAH patients with a mean age of 59 years and 65% (39 of 60) female patients. 85% (*n* = 51) patients suffered from anterior circulation aneurysms. 95% (*n* = 57) HH grade IV patients were included. 13.3% (*n* = 8) patients suffered from hydrocephalus at admission. 33.3% (*n* = 20) patients had developed cerebral edema at admission whereas 25% (*n* = 15) patients experienced midline shift at admission.

All patients were treated in the neurosurgical ICU according to standard treatment guidelines for aSAH. The clinical course was closely monitored with vital signs monitoring, electrocardiogram examinations, and serum electrolyte levels check. Bedside head CT was performed before and every 3 days after treatment to assess for complications including DCI. Furthermore, 50% (*n* = 30) patients were managed with the TTM protocol where the core body temperature was maintained between 36 °C and 37 °C before and during surgery, followed by rapid reduction to 34 °C for at least 72 h post-surgery. Afterwards, patients were slowly rewarmed to 36 °C to 37 °C at a rate of 0.1 °C per hour and kept at that temperature for at least 48 h.

All patients underwent definitive aneurysm treatment via endovascular embolization or microsurgical clipping within 24 h of disease onset. The choice between these two methods was made based on individual patient characteristics and aneurysm features. All parameters adopted to develop mRS predictive models for poor-grade aSAH patients are detailed. (Table 1)

**Table 1:** Parameters employed in the poor-grade aneurysmal subarachnoid hemorrhage patient dataset

Variables	Dataset
Total patients	60
**Demographics**	
Mean age (with SD)	59.1 (± 10.58) years
Gender	Male: 35% (*n* = 21) Female: 65% (*n* = 39)
Smoking history	Positive in 21.7% (*n* = 13) patients
Drinking history	Positive in 16.7% (*n* = 10) patients
Stroke history	Positive in 21.7% (*n* = 13) patients
Hypertension history	Positive in 46.7% (*n* = 28) patients
Diabetes history	Positive in 15% (*n* = 9) patients
Coronary disease history	Positive in 15% (*n* = 9) patients
**Laboratory parameters at day zero** ^**¶**^	
● **Complete blood count parameters**	
Red blood cell count	4.62 (± 2.25) x10^12^/L
White blood cell count	15.06 (± 4.85) x10^9^/L
Neutrophil count	87.2 (± 5.8) %
Lymphocyte count	9.25 (± 10.63) %
Neutrophil-to-lymphocyte ratio	15.55 (± 14.03)
Platelet count	224.08 (± 63.81) x10^9^/L
Platelet-to-White Blood Cell Ratio	16.35 (± 7.7)
Hemoglobin level	126.78 (± 18.73) g/L
● **Liver function parameters**	
Albumin level	39.53 (± 6.04) g/L
GPT level	26.72 (± 22.36) U/L
GOT level	36.32 (± 28.76) U/L
Total bilirubin level	13.94 (± 7.38) mol/L
Alkaline phosphatase level	78.96 (± 29.18) U/L
Glutamyl transpeptidase level	39.73 (± 61.6) U/L
● **Coagulation parameters**	
Prothrombin time	12.28 (± 1.73) s
Activated partial thromboplastin time	27.48 (± 6.45) s
● **Renal function parameter parameter**	
Creatinine level	57.42 (± 40.27) mol/L
**Radiological parameters at admission**	
Aneurysm location	Anterior circulation aneurysms in 85% (*n* = 51) patients
Cerebral edema	Positive in 33.3% (*n* = 20) patients
Midline shift	Positive in 25% (*n* = 15) patients
Hydrocephalus	Positive in 13.3% (*n* = 8) patients
Targeted temperature management	Adopted for 50% (*n* = 30) patients
**Functional mRS outcomes**	
mRS outcome at 1 month	Good outcome (mRS 0–3) in 21.7% (*n* = 13) patients
mRS outcome at 3 months	Good outcome (mRS 0–3) in 26.7% (*n* = 16) patients

¶ Laboratory parameters are provided as mean with standard deviation values

mRS = Modified Rankin Scale

### Prediction of mRS at 1 month

**Fig. 1:**
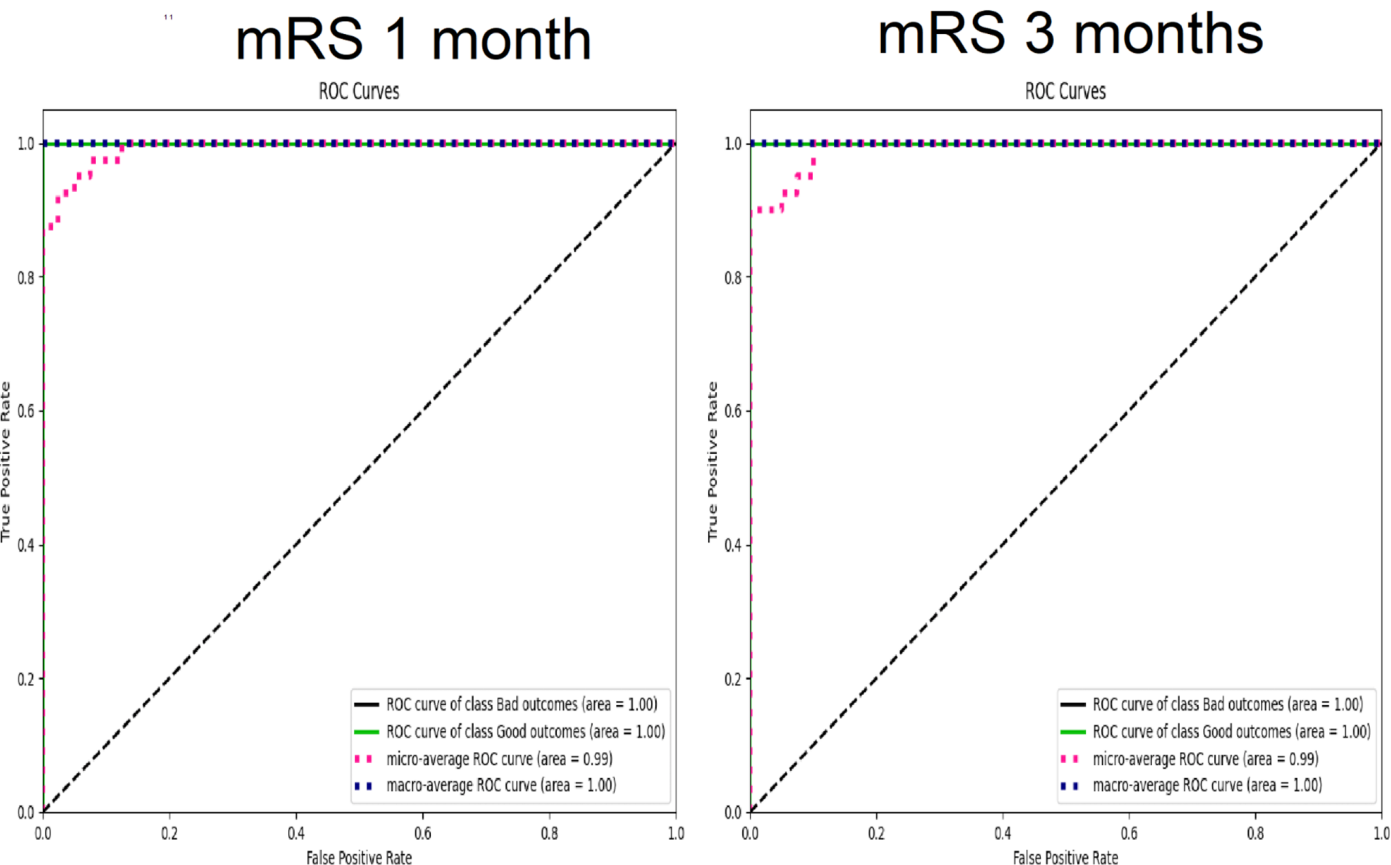
Area under the Receiver Operating Curve of the respective Logistic regression algorithmic models predicting modified Ranking scale (mRS) outcome at 1 month and 3 months in patients suffering from poor-grade aneurysmal subarachnoid hemorrhage

**Fig. 2:**
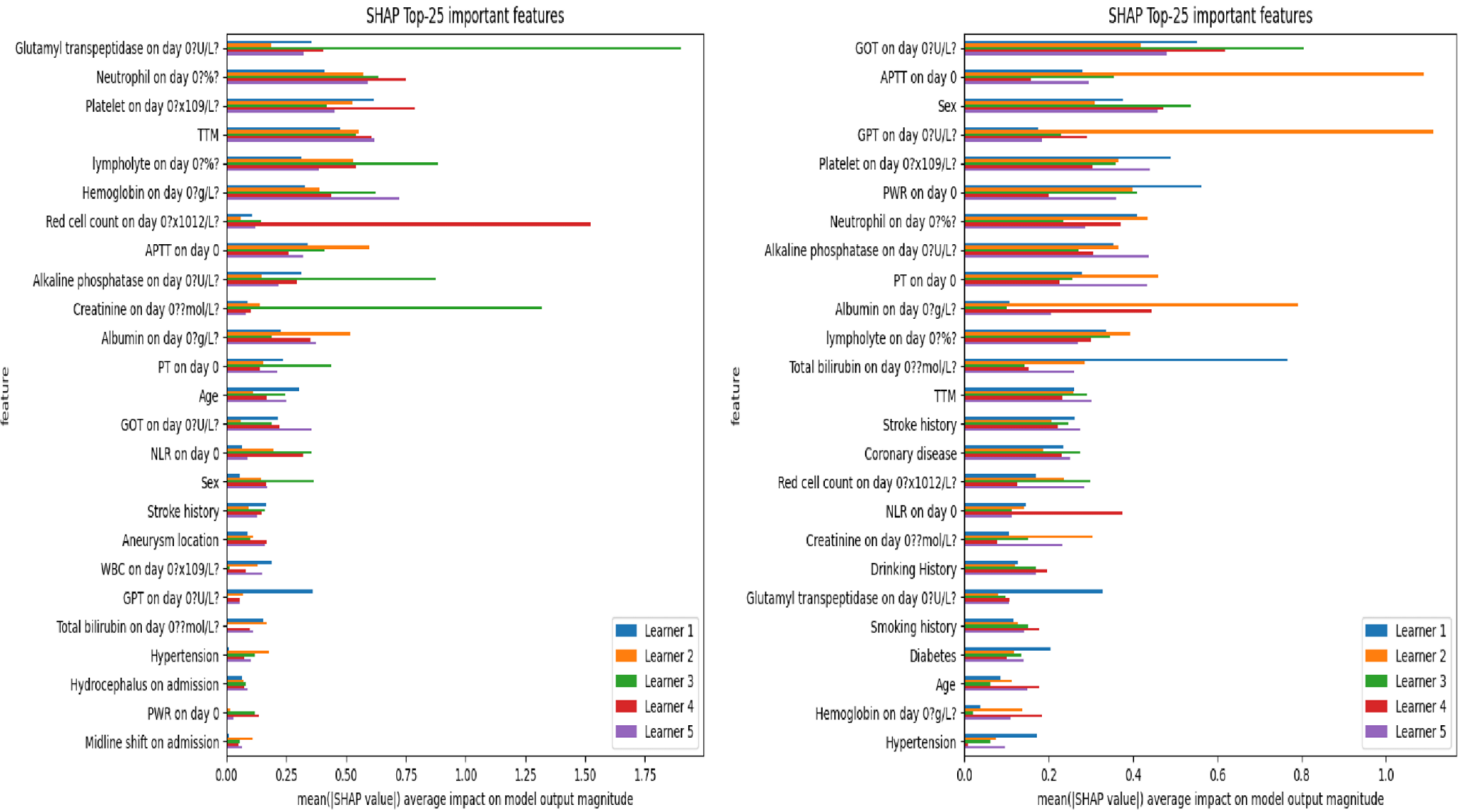
Influential predictors recognized by Logistic regression algorithmic model predicting modified Ranking scale outcome at 1 month (left) and 3 months (right) in patients suffering from poor-grade aneurysmal subarachnoid hemorrhage

**Fig. 3:**
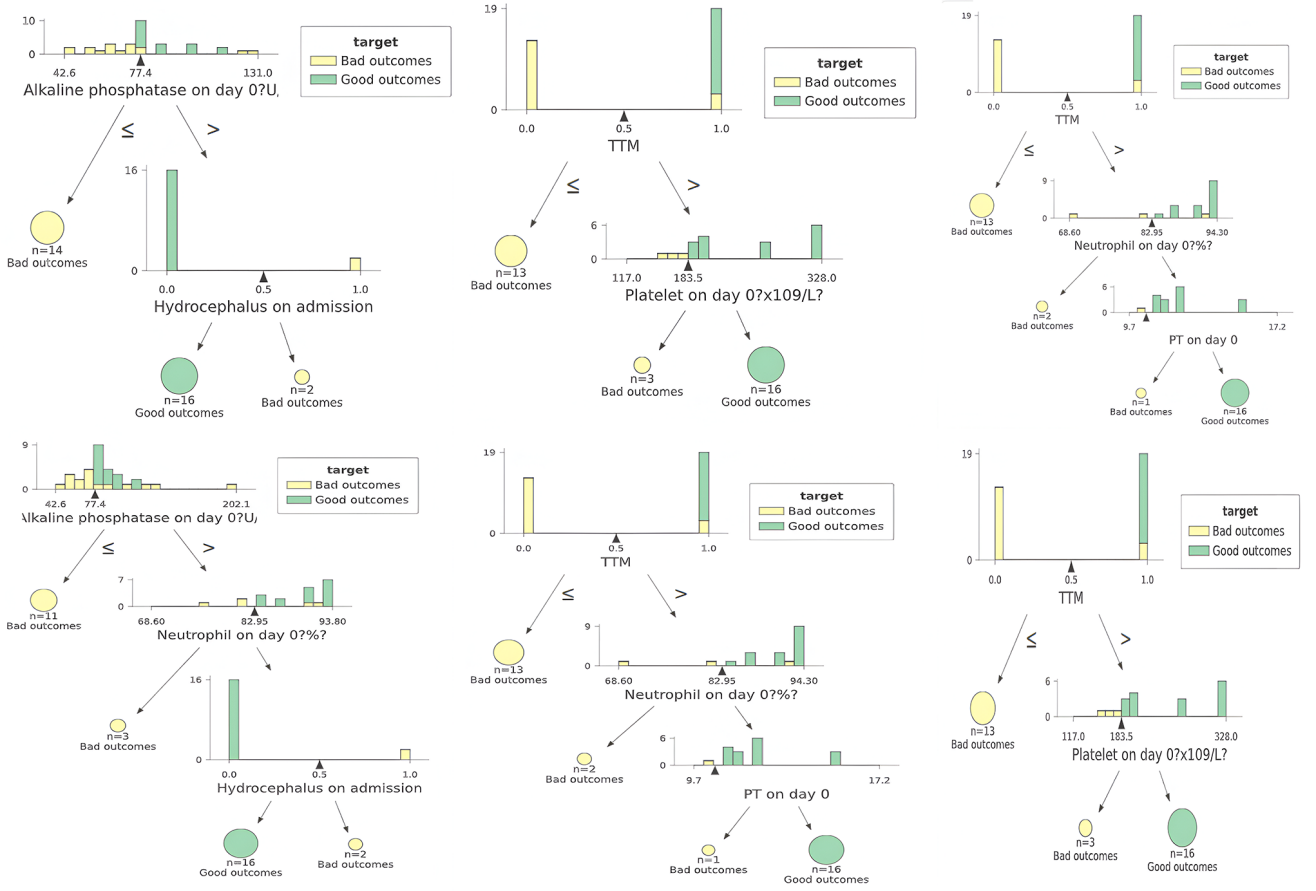
Rules delineated by Decision Tree algorithmic models predicting modified Ranking scale outcome at 1 month in patients suffering from poor-grade aneurysmal subarachnoid hemorrhage

Upon implementing the current SOTA for aML, a logistic regression (LR) algorithmic model predicted mRS at 1 month with 100% accuracy and an mWA-AUROC of 1. [F1-score = 100%, MCC = 1, Logloss = 0.18, Precision = 100%, Recall = 100%, F1-score = 100%, Sensitivity = 100% (95% CI: 83.16 − 100%), Specificity = 100% (95% CI: 83.16 − 100%). (Fig. 1) The model determined that the most significant predictors of mRS at one month were the neutrophil and platelet counts as well as gamma-glutamyl transferase (GTT) levels upon admission. (Fig. 2) Decision tree (DT) algorithmic models, all of which had an mWA-AUROC of 0.9, were able to reliably distinguish between good and poor mRS outcomes at 1 month by certain combinations of variables and cut-off points. Positive prognostic factors included ALP levels higher than mid-value of their normal range (higher than 77.4 IU/L), lack of hydrocephalus, use of TTM, excellent coagulation parameters including platelet levels higher than 183 × 109/L and prothrombin time (PT) higher than 9.7 s at day zero as well as immune inflammatory protective response (Neutrophil count higher than 82.95% of the white cell count at day zero) (Fig. 3).

### Prediction of mRS at 3 months

**Fig. 4:**
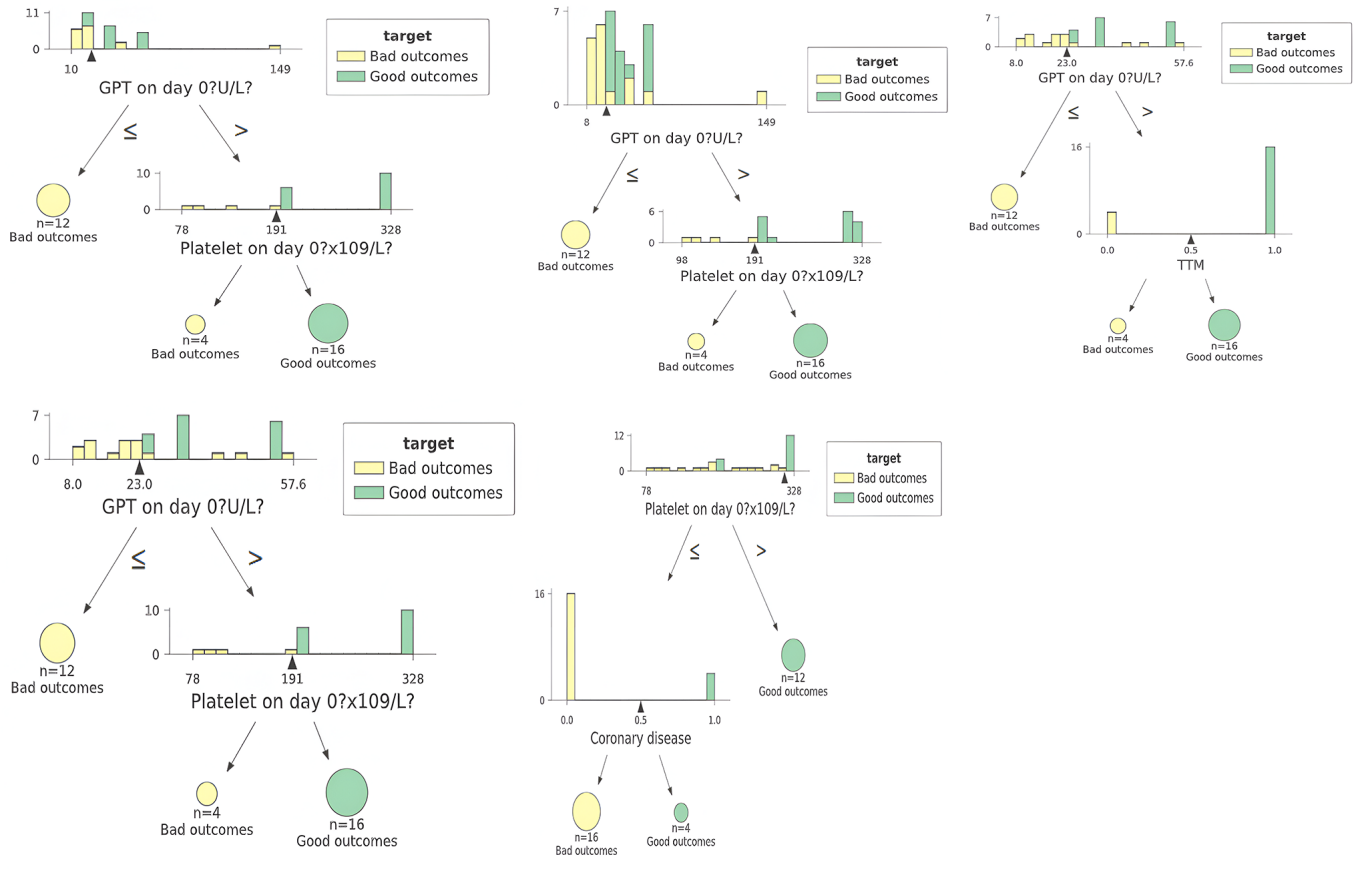
Rules delineated by Decision Tree algorithmic models predicting modified Ranking scale outcome at 3 months in patients suffering from poor-grade aneurysmal subarachnoid hemorrhage

An algorithmic LR model with an mWA-AUROC of 1 and 100% accuracy predicted mRS at 3 months. [F1-score = 100%, MCC = 1, Logloss = 0.16, Precision = 100%, Recall = 100%, F1-score = 100%, Sensitivity = 100% (95% CI: 83.16 − 100%), Specificity = 100% (95% CI: 83.16 − 100%). (Fig. 1) The most significant predictors of mRS at three months, according to the model, were the patient’s gender and the levels of activated partial thromboplastin time (aPTT) and serum glutamic oxaloacetic transaminase/aspartate aminotransferase (SGOT/AST) upon admission. (Fig. 2) DT algorithmic models, all of which had an mWA-AUROC of 0.925, were able to reliably distinguish between good and poor mRS outcomes at 3 months by certain combinations of variables and cut-off points. Strong favorable prognostic factors were GPT levels within the upper two-third of the normal range and platelet counts higher than 191 × 109/L at day zero (signifying hepatic and coagulation reserve), and the use of TTM. Furthermore, positive results were linked to the lack of coronary disease in patients with intermediate platelet counts (Fig. 4).

## Discussion

In this study, we adopted the SOTA for aML to develop algorithmic models that predicted mRS outcomes in patients suffering from poor-grade aSAH at 1 and 3 months using routinely measured laboratory and radiological parameters. It’s noteworthy that both time points achieved accuracy and mWA-AUROC of 1 which demonstrated the degree to which these algorithmic models captured complex interactions between clinical factors and outcomes.

Various combinations of variables were identified by the LR models to effect the 1-month and 3-month outcomes. GTT, neutrophil, and platelet counts were recognized as the most influential predictive factors at one month. These findings are consistent with a growing body of research that shows that inflammation and coagulation play an essential role in the initial brain damage that occurs after a SAH [26–28]. Elevated GTT levels might be an indication of oxidative stress or liver malfunction, both of which have been correlated with worsened outcomes in critical illness [29]. The potential impact of neuroinflammation and thrombo-inflammatory processes in the early stages of acute SAH is highlighted by the identification of neutrophil and platelet levels as important predictors [30]. Significant predictors for 3-month outcomes were patient gender, SGOT (AST), and aPTT levels. Gender-based differences in the outcomes of SAH have been reported [3]. The relevance of liver function tests (SGOT) in medium- and short-term prognoses points to a possible connection between neurological recovery and hepatic function in aSAH [31]. The significance of aPTT in predicting 3-month outcomes reinforces the rationale of using anticoagulation therapies for patients with aSAH, highlighting the long-lasting effects of coagulation abnormalities in aSAH [32]. 

The fluid nature of recovery following aSAH is demonstrated by the distinct predictors for the 1-month and 3-month outcomes [33]. Medium-term results appear to be determined by an amalgam of patient variables (gender) and sub-acute systemic abnormalities (liver function, coagulation status), whereas early outcomes are more impacted by acute physiological derangements (as evidenced by GTT and neutrophil counts) [8, 28]. The efficacy of therapies for patients with poor-grade aSAH might be influenced by the temporal progression of these prognostic factors [34]. 

Further insights were offered by the DT algorithms, which defined cut-off points for several factors that might direct clinical decision-making. In the initial period after poor-grade aSAH, there appears to be a complicated interaction between liver function, intracranial dynamics, and temperature control, as evidenced by the identification of ALP, hydrocephalus status, and TTM usage as significant predictors for 1-month outcomes [31]. The significance of GPT levels, platelet count, and neutrophil percentage for 3-month outcomes is consistent with our comprehension of the complex nature of aSAH recovery [33]. The defined cutoff points, such as platelet count > 191.0 × 10^9/L and GPT > 23.0-23.5 U/L, provide clinicians with precise, valuable particulars for aSAH prognosis and therapy planning [33]. Furthermore, the impact of TTM on improved outcomes suggests the likelihood of a neuroprotective impact, which may be stronger in particular subgroups [12]. This inference may make it easier to apply TTM to the aSAH patients who stand to gain the most, which might enhance patient outcomes and improve resource utilization [35]. 

The significance of coagulation measures (platelets, PT, aPTT) and inflammatory markers (neutrophils) in our models highlights the vital role that neuroinflammation and thrombo-inflammation play in the etiology of aSAH [26]. The impact of platelet count on the prognosis of aSAH has been explored [36]. Lee KS et al. found antiplatelet therapy to be associated with decreased probability of aSAH patients suffering from vasospasm and DCI with their use in the postictal state found to be associated with decreased in-hospital mortality and good functional outcomes [37]. Moreover, the neutrophil-to-lymphocyte ratio has been recognized as a predictor of aSAH outcomes, informing the role of neutrophils in the pathogenesis of aSAH [38, 39]. These results provided credence to the current investigation of tailored antithrombotic and anti-inflammatory treatments for the treatment of aSAH [40, 41]. 

The practical utility of our study is increased by its focus on clinical and laboratory indicators that are easily accessible at admission [42]. Early on in the course of therapy, the ability to make correct prognostications might have a substantial influence on clinical decision-making, resource allocation, and discussions regarding prognosis and care intensity with families [15]. Furthermore, the developed algorithmic models offered an objective, standardized approach to prognostication [43], which may be less subject to inter-observer variability compared to clinical observation [44, 45], especially in a low-resource setting where access to an experienced clinician is not readily available [46]. Especially, the developed algoirthmic models attempted to capture complex interactions associating various parameters, including liver enzymes and coagulation parameters, and outcomes that might not be immediately apparent through clinical observation alone. We, nevertheless, view the developed algorithmic models as a complement to, rather than a replacement for, ongoing clinical observation. The moels could provide an initial risk stratification, which clinicians can then refine based on the patient’s evolving clinical status.

Having said that, our study has a number of limitations. Although the modest sample size is adequate to illustrate the promise of ML techniques, it restricts the validity and applicability of our results [47]. Perfect predictive performance raises questions regarding possible overfitting [42]. Furthermore, even while it is feasible, our emphasis on variables easily obtainable on admission could not adequately reflect the dynamic changes that take place throughout the course of therapy in aSAH patients [8]. Incorporating variables pertinent to aneurysm (aneurysmal size and vasospasm) as well those encompassing management protocol (coiling vs. clipping, time to treatment, external ventricular drain placement for developing hydrocephalus) shall further add to the robustness of the resulting prognosticative model for it would capture longitudinal data [48–52].

Consequently, the main goal of future research should be to validate these models externally using larger, multi-center cohorts. Longitudinal data and treatment factors might be added to improve prediction accuracy and shed light on how different interventions affect results [47]. Refinement of these models and a deeper comprehension of the pathophysiology of aSAH might potentially be achieved by integrating neuroimaging data and molecular biomarkers [53]. To promote transparency and encourage further validation and development of our models, we have made our developed models and the associated architecture available at GitHub (https://github.com/hhaider15/Poor-grade-aSAH-mRS-outcomes-prediction.git*).* This shall allow other researchers and clinicians to easily implement and test our model, potentially accelerating the translation of these findings into clinical practice and facilitating the external validation process using larger, multi-center cohorts. A unique discovery that deserves more research is the identification of liver function tests (GTT, SGOT, and GPT) as important predictors across various time periods and algorithms for poor-grade aSAH outcomes. Although the liver is known to have a role in medication metabolism and systemic inflammation, its precise impact on neurological outcomes in aSAH remains unclear [54]. This finding suggests new avenues for investigation to establish a connection between brain recovery processes and liver function [55]. 

## Conclusion

Our research study elucidated how well aML can predict functional outcomes in patients with poor-grade aSAH. Through the identification of novel indicators and their interplay, these models illuminated possible targets for optimized treatment. This aML-powered approach is an important advancement toward tailored therapy in the treatment of this difficult ailment, even though additional validation is required. The availability of our algorithmic models as a Google Colab notebook shall facilitate its implementation and further validation, potentially accelerating the integration of ML-based prognostication into clinical practice for poor-grade aSAH patients. Efforts should be undertaken to improve care and outcomes for patients with poor-grade aSAH by refining these models and integrating them with clinical expertise.

## Data Availability

The dataset adopted for this study, the associated Google Colab notebook and the developed algorithmic models are accessible on a public GitHub repository (https://github.com/hhaider15/Poor-grade-aSAH-mRS-outcomes-prediction.git).
